# Identification of EVC variants and the preimplantation genetic testing in a Chinese family

**DOI:** 10.3389/fmed.2026.1798913

**Published:** 2026-05-11

**Authors:** Haiyan Luo, Zhen Guo, Yao Yu, Jia Chen, Qing Lu, Yan Yang, Ting Huang, Danping Liu, Wangli Nie, Wan Lu, Bicheng Yang, Jun Zou, Yanqiu Liu, Yongyi Zou

**Affiliations:** 1 Jiangxi Maternal and Child Health Hospital, Nanchang, Jiangxi, China; 2 Jiujiang Polytechnic University of Science and Technology, Gongqing, Jiangxi, China; 3 Jiujiang Maternal and Child Health Hospital, Jiujiang, Jiangxi, China

**Keywords:** EVC, EVC syndrome, preimplantation genetic testing, prenatal diagnosis, WES

## Abstract

Fetal genetic skeletal disorders are common congenital anomalies with notable genetic and phenotypic heterogeneity. Genetic analysis plays an important role in the definitive diagnosis of these skeletal conditions. In the present study, we aimed to determine the causative genetic factors underlying fetal skeletal disorders with shortened limbs (femoral lengths <−2.0 standard deviations) detected by routine second-trimester prenatal ultrasonography in a Chinese family. Whole exome sequencing (WES) analysis identified *in trans* heterozygous variants in EVC in the affected fetus, including a previously unreported frameshift variant (NM_153717.2: c.130del) and a previously reported variant (NM_153717.2: c.1099_1563del). Combined genetic and clinical information indicated a diagnosis of Ellis-van Creveld (EVC, OMIM 225500) syndrome in the affected fetus. The functional impact of this previously unreported variant on mRNA and protein expression was assessed *in vitro* using quantitative reverse transcription PCR (RT-qPCR) and Western blot analysis. *In vitro* RT-qPCR analysis revealed that the c.130del variant did not affect the transcriptional expression of the EVC gene, suggesting the likely absence of nonsense-mediated mRNA decay (NMD). In contrast, Western blot analysis demonstrated the accumulation of truncated protein isoforms, indicating a potential role in the pathogenic mechanism. Preimplantation genetic testing (PGT) was performed on this family for a baby not affected by EVC syndrome or chromosomal aneuploidies. The present study identified and characterized a previously unreported variant in the EVC gene, which has broadened the established mutational spectrum and held notable implications for the clinical management and genetic counseling of families affected by EVC syndrome.

## Introduction

Genetic skeletal dysplasia represents a heterogeneous group of genetic disorders. The overall incidence of these conditions is estimated at ~1/5,000 births, accounting for nearly 10% of prenatal mortality (1). Ellis-van Creveld (EVC) syndrome (OMIM 225500) is an autosomal recessive congenital skeletal ciliopathy with an estimated incidence of ~1/60,000 live births in the general population. Notably, the prevalence is markedly higher in the Old Order Amish population of Lancaster County (Pennsylvania, United States), reaching ~5/1,000 live births (2–5). EVC syndrome demonstrates substantial genetic heterogeneity, with ~70% of the cases resulting from nonsense or frameshift variants and leading to loss-of-function alterations in either the EVC (OMIM *604831) or EVC2 (OMIM *607261) gene (6).

Postnatally, Ellis-van Creveld (EVC) syndrome is classically defined by a recognizable constellation of skeletal anomalies. These include disproportionate short stature with predominant rhizomelic limb shortening, a characteristic narrow and elongated thorax associated with shortened ribs, and postaxial polydactyly affecting one or both hands and/or feet, frequently accompanied by nail dysplasia (7, 8). Fetal structural abnormalities of EVC syndrome, particularly limb shortening can be detected as early as week 15 of gestation. However, additional anomalies—such as a narrow thorax, postaxial polydactyly of the hands, and cardiac defects—are typically detectable only during anomaly scans performed in the late second or third trimester (3, 9–11). The lack of a family history, together with the nonspecific and subtle sonographic features of EVC syndrome on routine second-trimester ultrasound, poses notable challenges for conclusive diagnosis during the early stages. This often leads to considerable psychological distress for expectant parents and increases the maternal risks when late-term pregnancy termination is carried out. Therefore, the integration of genetic testing with the sonographic detection of shortened limbs is crucial for the early and conclusive diagnosis of EVC syndrome in fetuses. Recent breakthroughs in next-generation sequencing (NGS) technologies have brought high-throughput genomic interrogation approaches, especially whole exome sequencing (WES), to the forefront of clinical diagnostics (12–14). Growing evidence from large-scale cohort studies indicates that WES attains a diagnostic yield ranging 65.8–83.3% in the prenatal diagnosis of fetal skeletal system abnormalities. These findings underscore the clinical value of WES in perinatal genetic diagnosis (15–17).

To date, a total of 72 distinct pathogenic variants in the EVC gene have been documented as being linked to EVC syndrome in the professional Human Gene Mutation Database.^1^ Despite the advancements made in molecular profiling, only nine disease-associated variants in EVC have been well-characterized within Chinese populations to date (18–23). This limited genetic understanding highlights the critical need for expanded research to fully elucidate the mutational spectrum of EVC within this demographic. Such efforts are crucial for establishing robust frameworks to support precision genetic counseling and evidence-based clinical management. In the present study, a previously unreported frameshift variant EVC c.130del identified in a Chinese fetus is presented, which has further broadened the mutational spectrum of EVC syndrome.

## Materials and methods

### Subject and clinical information

**Figure 1:**
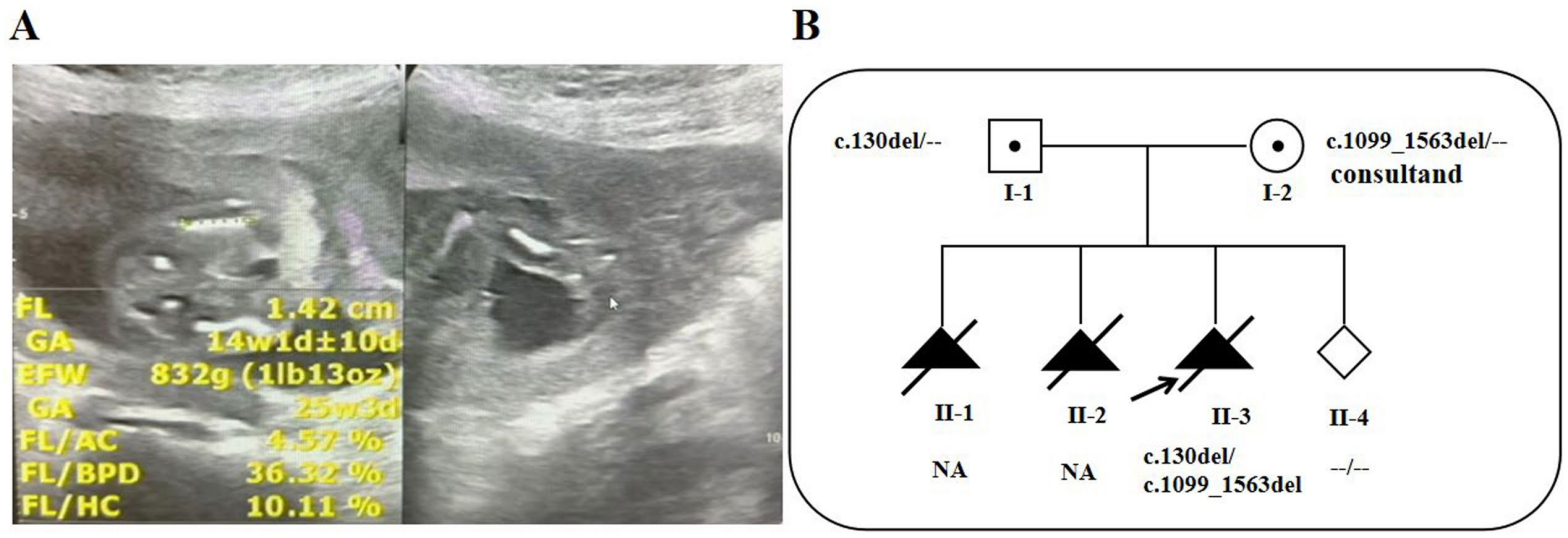
**(A)** Markedly shortened long bones (<−2.0 SD) by sonographic examination at 16 + 5 weeks’ gestation. **(B)** An unfilled diamond denotes a fetus of undetermined sex, while a filled triangle indicates a pregnancy that was selectively terminated. I-1: father of the proband; I-2: mother of the proband; II-1 and II-2: spontaneous abortions; II-3: the proband; II-4: the embryo conceived through PGT. “NA”: Genotype information is unavailable.

The participant was a 28-year-old G3P0 (three pregnancies and zero live births) woman at week 16 of gestation with a spontaneously conceived pregnancy. The patient denied consanguinity and reported a history of two previous pregnancies that were terminated due to fetal abnormalities. The patient’s first pregnancy was complicated by shortened limb hypoplasia detected at 16 + 4 weeks of gestation, while her second pregnancy was complicated by both shortened limb hypoplasia and a cardiac malformation identified at 28 + 5 weeks. Both pregnancies were terminated owing to the presence of the aforementioned significant fetal structural anomalies, and no other anomalies were noted. However, no prenatal genetic testing was performed to confirm the diagnosis, and no archived fetal tissue specimens were available for retrospective analysis after either pregnancy termination. The patient was in her third pregnancy, which had been unremarkable during routine prenatal visits. At 16 weeks of gestation, a routine second-trimester ultrasound performed for fetal developmental assessment revealed sonographic findings suggestive of disproportionate fetal limb shortening. A follow-up examination at 18 + 1 weeks of gestation confirmed these findings, demonstrating a persistent disproportionate limb shortening pattern. Collectively, these two consecutive prenatal ultrasound examinations identified fetal skeletal abnormalities characterized by shortened limbs, with femoral lengths falling more than 2.0 standard deviations below the gestational age-specific mean. Comprehensive clinical parameters associated with these findings are presented in Table 1. Prenatal ultrasonography demonstrated features indicative of fetal skeletal disorder (Figure 1A), and a schematic pedigree of the family was presented in Figure 1B. The collection of all biological specimens and clinical data was conducted following the acquisition of written informed consent from all participants. The present study was approved by the Ethics Committee of Jiangxi Maternal and Child Health Hospital (Nanchang, China) (No. 20210324).

**Table 1:** Assessment of fetal clinical parameters by two consecutive ultrasonographic examinations.

No.	GA (W)	BPD (mm)	HC (mm)	AC (mm)	HL (mm)	FL (mm)	Clinical evaluation
1	16 W	31	114	109	12.6	10.7	HL: −1.57 SD FL: −2.02 SD
2	18 W + 1	39	141	118	16.0	14.0	HL: −1.33 SD FL: −2.85 SD

GA, gestational age; BPD, biparietal diameter; HD, head circumference; AD, abdomen circumference; HL, humerus length; FL, femur length, SD: standard deviation.

### WES and the integrated bio-informatics pipeline

WES on the amniocytes was conducted by Illumina, Inc., with an average depth of 200x, covering 95% of the target regions. Genomic DNA was fragmented using a S220 focused-ultrasonicator (Covaris, LLC) and subsequently enriched using the SureSelect Human All Exon V6 Kit (Agilent Technologies, Inc.). Paired-end libraries were sequenced on an Illumina HiSeq 2,500 platform (Illumina, Inc.), generating 101-bp paired-end reads. Quality-controlled sequencing reads were aligned to the human reference genome (hg19/GRCh37) using NOVOAlign. Variant calling was performed using the Genome Analysis Toolkit. Variants identified were subsequently filtered to exclude those with a minor allele frequency >0.001 in either the Genome Aggregation Database or the Chinese Exome Sequence Database. Synonymous variants without predicted splicing effects were also removed. Deleterious single-nucleotide variants were prioritized using multiple prediction algorithms, including SIFT, PolyPhen-2, MutationTaster and PROVEAN. Potential inherited variants and *de novo* variants associated with skeletal dysplasia were then screened using the MIRTrios program.

### Validation of causative EVC variants identified by WES

The causative variants primarily identified by WES were further validated by Sanger sequencing or RT-qPCR. Sanger sequencing was performed on an Applied Biosystems 3,500 DX Genetic Analyzer (Thermo Fisher Scientific, Inc.) and the resulting chromatograms were aligned and compared against the reference transcript sequence. Exon-level deletions or duplications by RT-qPCR were quantified in a Bio-Rad iCycler real-time PCR detection system (Bio-Rad Laboratories, Inc.) using SYBR Green I dye, with a specific primer pair for each exon. To normalize for potential variations in DNA input or the presence of PCR inhibitors, the reference gene *HBB* was amplified and quantified in parallel reactions for each sample. Genomic DNA was quantified at a concentration of 100 ng/μL for RT-qPCR, and all experiments were performed in three technical replicates. The sequences of the primers used for PCR are provided in Table 2.

**Table 2:** Primers used in the study.

Primers	Primer length (bp)	Sequence (5′-3′)
EVC-EX1-F	20	AAGGGGAGAGAAGCAGGAGT
EVC-EX1-R	20	TGTCAAGGATCACCCAACGG
EVC-EX9-F	20	CAGCTATGGTCCTGTGTGTT
EVC-EX9-R	20	CTGGACTTGCAGCTTCAGAA
EVC-EX10-F	19	ATCCCAGAGTTTGTCCAGCG
EVC-EX10-R	20	CTCTGTTCCTCCTCTTGGGC
EVC-EX11-F	20	AGGAGAATGTCAGAGCCACC
EVC-EX11-R	20	GTTCTCGCACACACACAGAC
HBB-EX2-F	21	TGACTCTCTCTGCCTATTGGTCTA
HBB-EX2-R	24	TTAGGGTTGCCCATAACAGCA

### Construction of expression vectors and cell transfection

The recombinant vector pEGFP-C1 EVC wt was constructed by inserting the full-length human EVC complementary DNA (cDNA) into the pEGFP-C1 plasmid using HindIII and KpnI restriction sites for subsequent expression. The c.130del (p.Leu44Phefs*72) variant was subsequently introduced into this vector by site-directed mutagenesis (Baiyi Biopharm Co., Ltd.) to generate the mutant construct pEGFP-C1 EVC mut. Both the wild-type and mutant eukaryotic expression vectors were transiently transfected into HEK293T cells using Lipofectamine 2000.

### *In vitro* RNA analysis and western blotting

Total RNA was extracted from cell samples 48 h after transfection using the Trizol reagent (Invitrogen; Thermo Fisher Scientific, Inc.). cDNA was synthesized in accordance with the manufacturer’s protocol (Takara Biotechnology Co., Ltd.). The mRNA expression levels of wild-type and mutant target genes were then analyzed by RT-qPCR on an ABI StepOne Real-Time PCR system (Applied Biosystems) using SYBR Green Realtime PCR Master Mix (LMAI Bio). At 36 h post-transfection with wild-type or mutant recombinant expression vectors, total protein was extracted from cell pellets using RIPA lysis buffer supplemented with protease and phosphatase inhibitors (Servicebio). For Western blot analysis, protein concentrations were determined using a BCA protein assay kit (Yeasen). Equal amounts of protein (20 μg per lane) were resolved by 12.5% SDS-PAGE and electrotransferred onto PVDF membranes (Bio-Rad). The membranes were blocked with 5% non-fat milk in TBST for 1 h at room temperature, followed by overnight incubation at 4 °C with mouse monoclonal primary antibodies against Flag (clone 2064, DAIAN, 1:1,000), GFP (clone 2057, DAIAN, 1:1,000), and GAPDH (GB15002, Servicebio, 1:1,000). Here, GFP served as a transfection/expression control, while GAPDH was used as a loading control. After washing with TBST, the membranes were incubated with an HRP-conjugated goat anti-mouse IgG secondary antibody (Proteintech, 1:20,000) for 1 h at room temperature. Chemiluminescent signals were detected using an ECL substrate (Servicebio) and visualized with a QuickChemi 5,100 imaging system (Monad). Densitometric analysis was performed using ImageJ software. All experiments were performed in three independent biological replicates (*n* = 3). For each biological replicate, three technical replicates were measured to assess intra-assay variability.

### PGT analysis

NGS-based single nucleotide polymorphism (SNP) haplotyping was performed on the prospective parents and an affected family member to delineate the chromosomal haplotypes associated with the normal and disease-associated haplotypes. The target gene, along with >200 informative SNPs within 2 Mb range were sequenced by NGS (Peking Jabrehoo Med-Tech Co., Ltd) to identify disease-associated and normal haplotypes within the family. Only heterozygous SNPs were considered informative for haplotype phasing. Subsequently, blastocysts that survived until day 5 underwent laser-assisted hatching and trophectoderm biopsy. The biopsied samples were subjected to whole-genome amplification via multiple displacement amplification and whole genome amplification. After which, the amplified products were used for comprehensive genomic analyses, including SNP haplotyping, Sanger sequencing, aneuploidy screening and copy number variation analysis. Embryos with the normal SNP haplotypes identified by NGS were selected and further confirmed by Sanger sequencing. Additionally, low-coverage whole-genome NGS was employed to detect chromosomal aneuploidies and structural imbalances >1 Mb for PGT for aneuploidy. Ultimately, only embryos negative for two causative variants in EVC and free of chromosomal aneuploidies were considered suitable for transfer. Amniocentesis was subsequently performed to validate the genetic results of the PGT.

### Statistical analysis

Statistical analysis was performed using GraphPad Prism 8 software. Measurement data were presented as the mean ± standard deviation. Differences among two groups were compared using independent samples *t*-test. *p* < 0.05 was considered to indicate a statistically significant difference.

## Results

### Variant interpretation and annotation

WES identified *in trans* heterozygous variants, NM_153717.2: c.130del (p.Leu44Phefs*72, inherited paternally) and NM_153717.2: c.1099_1563del (exon 9 to exon 11, inherited maternally) in EVC as the primary findings. Additionally, the secondary findings reported four heterozygous variants: NM_001853.3: c.413G > A (p.Arg138His, inherited paternally) in *COL9A3*, NM_001456.3: c.1141G > A (p.Ala381Thr, inherited maternally) in *FLNA*, NM_004994.2: c.1232A > T (p.His411Leu, inherited paternally) in *MMP9* and NM_004560.3: c.1687G > A (p.Glu563Lys, inherited maternally) in *ROR2*. Both EVC variants are absent from the gnomAD database. *In silico* analysis using the LUMC Mutalyzer 3 tool predicted that these variants result in the loss of a substantial portion of the protein. The unreported variant c.130del (PVS1+PM2_Supporting) and the previously documented c.1099_1563del (PVS1_Strong+PM2_Supporting+PP3) in EVC were both classified as pathogenic according to the ACMG/AMP guidelines (PVS1 + PM2_supporting+PM3) (24). The remaining four heterozygous variants were classified as variants of uncertain significance based on the same guidelines. No additional family members beyond the parents were available for further genetic testing. Based on the correlation between the fetal phenotypes and co-segregation analysis within the family, we propose that the compound heterozygous variants in EVC are the likely causative genetic factors underlying the observed skeletal anomalies in the affected fetus.

### Validation of two EVC variants by Sanger sequencing and RT-qPCR

**Figure 2:**
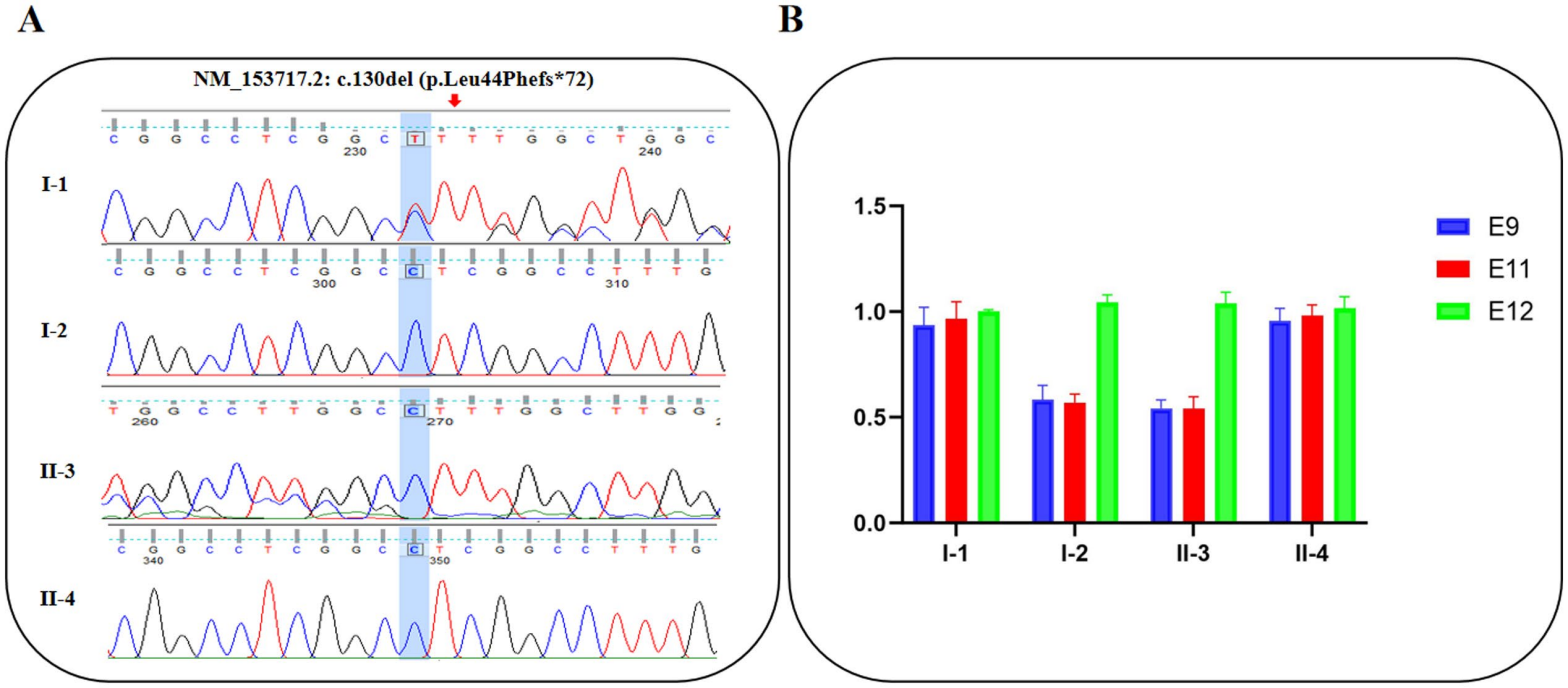
**(A)** DNA sequence analysis of the c.130del variant in the pedigree. **(B)** Copy number analysis of exon 9 to 11 in EVC gene by RT-qPCR performed on genomic DNA in the pedigree.

Point variant and multi-exon deletion analyses in EVC were confirmed using Sanger sequencing and RT-qPCR, respectively (Figures 2A,B). Sanger sequencing confirmed the presence of c.130del in the proband and the father, whereas the mother carried the wild-type allele at this locus. RT-qPCR analysis of genomic DNA revealed an ~50% reduction in the copy number of exons 9–11 in both the proband and the mother compared with the father, which was consistent with the findings from WES.

### *In vitro* mRNA analysis and WB

**Figure 3:**
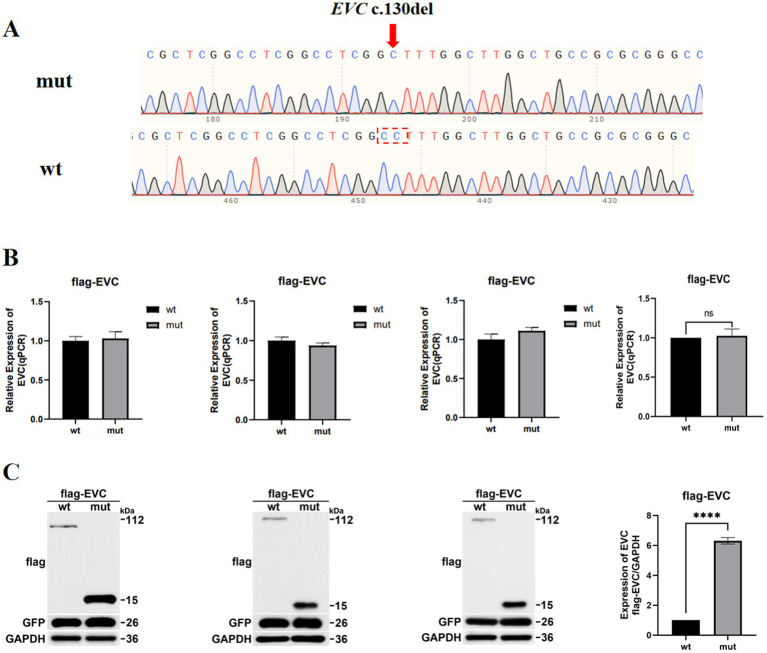
Functional analysis of the c.130del variant in HEK293T cells. **(A)** Sequencing confirmation of the c.130del variant. **(B)** RT-qPCR analysis showing no significant difference in EVC mRNA expression between mutant and wild-type cells. **(C)** Western blot analysis performed in three independent biological replicates in the wildtype and the EVC-mutant (mut) HEK293T cells.

The constructed eukaryotic expression vectors for wild-type (pEGFP-C1 EVC wt) and mutant (pEGFP-C1 EVC c.130del, p.Leu44Phefs*72) were validated by Sanger sequencing (Figure 3A). RT-qPCR analysis showed no significant difference in the mRNA expression levels of the c.130del variant relative to the wild-type control in HEK293T cells (Figure 3B), indicating that this variant does not markedly alter EVC transcript levels in this *in vitro* system. However, western blot analysis demonstrated that the c.130del variant introduces a premature termination codon, resulting in a truncated protein (GFP-EVC-mut1, ~15 kDa) instead of the full-length product (GFP-EVC-wt, ~112 kDa) (Figure 3C). Under the present experimental conditions, the truncated protein accumulated to markedly higher steady-state levels than the wild-type protein. The quantified difference in protein expression between the mutant and wild-type groups was 5.31 ± 0.22 (mean±SEM).

### PGT and prenatal diagnosis

**Figure 4:**
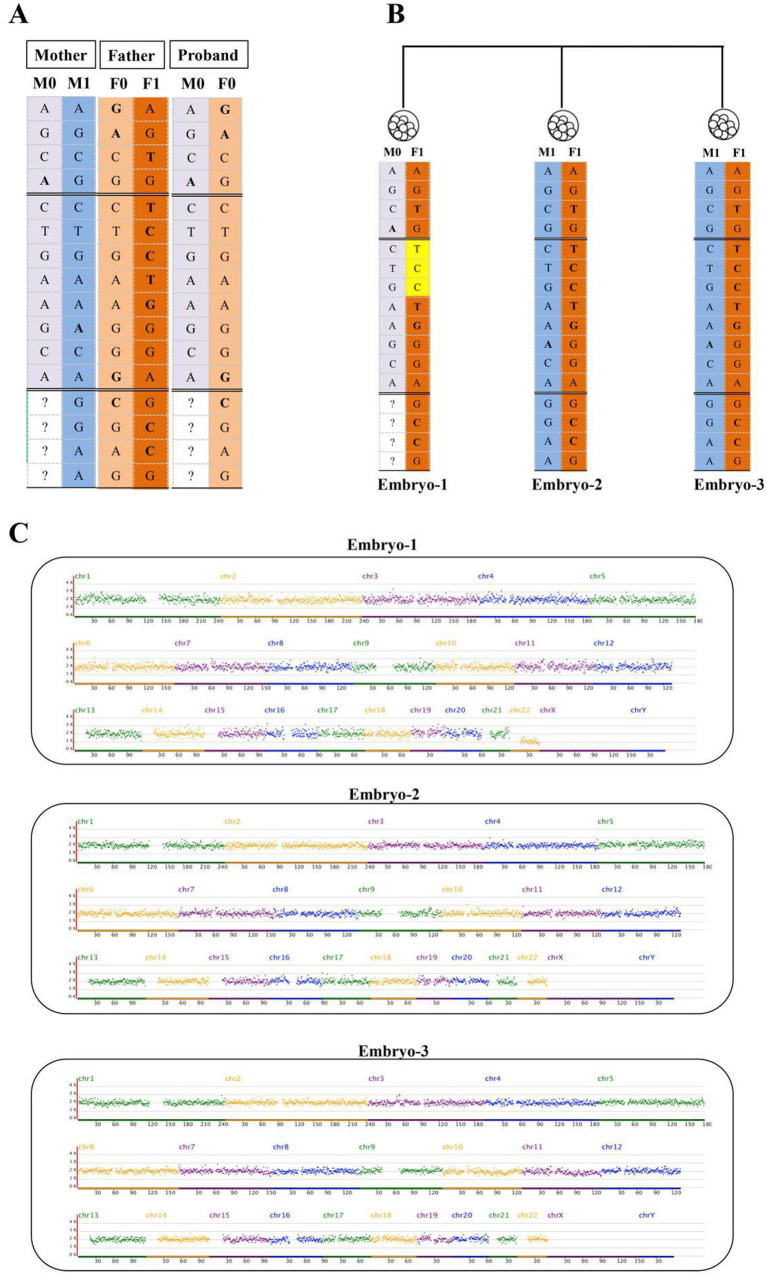
**(A)** Haplotype-based linkage analysis performed for the EVC gene. M0 and F0: The maternal and paternal at-risk alleles on chromosome 4; M1 and F1: The corresponding non-risk alleles. **(B)** The SNP-haplotype linkage analysis for the three embryos. **(C)** Screening results of the PGT-A. Information regarding sex chromosomes is omitted in compliance with regulations governing maternal and infant health care in mainland China.

In the PGT cycle, three embryos developed to the blastocyst stage and underwent trophectoderm biopsy. Genetic analysis yielded conclusive results for all three embryos. Haplotype linkage analysis revealed that Embryo 1 carried the maternal at-risk SNP haplotype, indicating the presence of the heterozygous NM_153717.2: c.1099_1563del associated with EVC syndrome. Embryos 2 and 3 carried maternal and paternal wild-type haplotypes and were therefore genetically unaffected by EVC syndrome (Figures 4A–C). Concurrent PGT for aneuploidy analysis demonstrated that Embryo 1 was monosomic for chromosome 22 (45,XN,-22), whereas Embryos 2 and 3 exhibited a euploid karyotype. Following comprehensive genetic screening, Embryo 3 was prioritized for transfer based on its euploid status and the absence of causative EVC variants. Subsequent prenatal diagnosis confirmed a normal fetal genotype without genomic aberrations. A healthy male infant was born and has since remained well, with normal findings on all routine physical examinations during consistent follow-up to date.

## Discussion

Since the initial identification of the EVC gene in the year 2000, homozygous or *in trans* heterozygous variants in EVC have been established as the genetic cause of EVC syndrome (25). To date, a variety of EVC variants, predominantly nonsense or frameshift variants, have been identified in individuals with EVC syndrome (26). Nevertheless, the spectrum of variants characterized in the Chinese population still remains limited. In the present study, a novel variant NM_153717.2: c.130del and a previously reported variant NM_153717.2: c.1099_1563del were identified in an affected Chinese fetus. The present study reports the 10th EVC variant linked to EVC syndrome in the Chinese population. In the present study, *in vitro* experiments demonstrated that the variant c.130del did not impair transcriptional activity, suggesting the likely absence of nonsense-mediated mRNA decay (NMD). In contrast, Western blot analysis revealed the accumulation of truncated protein isoforms in vitro, which may contribute to the disease mechanism. However, it is important to note that overexpression experiments in HEK 293 T cells carry inherent limitations, as this heterologous system may not fully recapitulate the tissue-specific biological context of EVC function. Given the rarity of the disorder and the paucity of reported cases, further genetic validation in independent cohorts and functional assays in more physiologically relevant models are essential to definitively establish variant pathogenicity and to elucidate the precise molecular mechanisms involved.

EVC syndrome contributes to a broad spectrum of phenotypic variability and involves multiple organ systems (27). Postnatally, cardinal clinical features include short limbs and ribs, postaxial polydactyly, hypodontia, hypoplastic nails, fusion of the capitate and hamate bones, cone-shaped epiphyses of the phalanges, bowing of the humerus and proximal tibiofibular fusion (3). Nevertheless, fetal phenotypes can be relatively nonspecific, and overt manifestations often do not become apparent until the third trimester, which poses a challenge for prenatal ultrasound evaluation and differential diagnosis in the early stages (28, 29). Therefore, definitive prenatal diagnosis of EVC syndrome by ultrasound evaluation is often hindered by limited information on comprehensive and characteristic fetal phenotypes in the early second-trimester. Consequently, while there is extensive literature on the postnatal diagnosis of EVC syndrome, dedicated clinical studies on its prenatal diagnosis remain limited. In the present case, routine fetal ultrasonography performed at 16 + 5 weeks gestation revealed only the cardinal manifestation of short limbs in EVC syndrome, which may be attributable to the relatively early gestational age and routine scanning methodology. However, a definitive diagnosis of fetal EVC syndrome in the second trimester was established based on genetic analysis and subtle ultrasonographic findings. To the best of our knowledge, the present study represents. To the best of our knowledge, this case represents one of the earliest genetically confirmed prenatal diagnoses of EVC syndrome, achieved in the early second trimester. This finding extends the window for prenatal diagnosis of EVC syndrome to the early second trimester.

PGT has emerged as a pivotal technique in assisted reproductive technology, contributing to the achievement of successful pregnancies. Consequently, it has gained widespread adoption as a diagnostic tool in recent clinical practice (30, 31). PGT is categorized into three distinct subtypes, namely PGT for aneuploidies, for monogenic disorders and for structural rearrangements. It has now become a well-established alternative to conventional invasive prenatal diagnostic procedures (32, 33). The present study reported on a severe familial case involving three consecutive pregnancies complicated by fetal skeletal anomalies, which was confirmed as EVC syndrome by combined genetic diagnosis and subtle ultrasonographic findings at the second trimester. PGT was recommended to this family and brought the birth of a healthy male infant. This case highlights the clinical utility and feasibility of PGT, underscoring its potential for broader application in effectively preventing the recurrence of specific genetic disorders.

In summary, a novel pathogenic variant in EVC was identified in a Chinese fetus. The findings of the present study broadened the established mutational spectrum of EVC syndrome and holds notable implications for the clinical management and genetic counseling of families affected by EVC.

## Data Availability

The datasets presented in this study can be found in online repositories. The names of the repository/repositories and accession number(s) can be found in the article/supplementary material.

## Footnotes

1
http://www.hgmd.cf.ac.uk/ac/all.php
